# Immunotoxin Complementation of HAART to Deplete Persisting HIV-Infected Cell Reservoirs

**DOI:** 10.1371/journal.ppat.1000803

**Published:** 2010-06-10

**Authors:** Edward A. Berger, Ira Pastan

**Affiliations:** 1 Laboratory of Viral Diseases, National Institute of Allergy and Infectious Diseases, National Institutes of Health, Bethesda, Maryland, United States of America; 2 Laboratory of Molecular Biology, National Cancer Institute, National Institutes of Health, Bethesda, Maryland, United States of America; The Fox Chase Cancer Center, United States of America

## Achievements and Limitations of Antiretroviral Therapy

The development of combinations of drugs that potently suppress HIV replication, collectively given the acronym HAART (highly active antiretroviral therapy), has transformed the lives of people with HIV infection, particularly in high-income countries [1]. Though modern HAART regimens can drive HIV plasma viral loads below the detection limits of standard clinical assays (≥50 copies HIV RNA/ml), long-term treatment fails to eradicate infectious virus as revealed by the persistence of HIV proviral DNA and infectious HIV in peripheral blood and lymphoid tissue, as well as by low level viremia (1–50 RNA copies/ml) in the majority of treated people as detected by ultrasensitive single copy assays [2]–[4]. Moreover, reservoirs of latently infected resting memory CD4^+^ T lymphocytes are established early after infection and persist throughout treatment with exceedingly slow decay rates; these latent reservoirs are unlikely to be eliminated by HAART alone, and thus have the potential to re-ignite the infection if activated after therapy is halted. A further complication is the existence of multiple sanctuaries of infection in cell types from various lineages (monocyte-macrophages, dendritic cells, hemapoietic stem cells, etc.) detected in distinct anatomical compartments (blood, peripheral lymph nodes, gut mucosa, central nervous system, genital tract, etc.). These findings raise a number of critical inter-related questions: Does the extreme stability of the latently infected cell reservoirs reflect simply the long intrinsic half-life of memory CD4^+^ T lymphocytes, and/or are the reservoirs continuously reseeded by low level ongoing replication? To what extent does residual viremia reflect incomplete suppression of replication versus virus output from stable (perhaps renewable) infected cell reservoirs? What is the source(s) and significance of intermittent viremia blips, and from where does HIV rebound upon cessation of HAART? Will deliberate activation of resting CD4^+^ T lymphocytes under continued HAART provide a clinical benefit by depleting latently infected cell reservoirs? While these issues remain controversial, a major practical consequence is irrefutable: cessation of HAART results in rapid virus rebound, in many cases to pre-treatment levels. As a result, treatment must be long-term, presumably for life.

## A Renewed Focus on HIV Eradication

The profound viral suppression achievable with modern-day HAART regimens coupled with the limitations and concerns of prolonged treatment (cumulative side effects, adherence difficulties, emergence of drug resistance, high costs) have revitalized serious consideration of the prospect for eradicating HIV from the body, or at least of achieving a “functional cure” whereby therapy can be stopped without viral rebound [3]–[9]. The latently infected CD4^+^ T cell reservoirs have generally been viewed as the major obstacle to eradication; hence there has been considerable focus on therapeutic strategies to drive the proviral genome out of latency, including cytokines (e.g., IL-2), histone deacetylase inhibitors (e.g., valproic acid, SAHA), nontumorogenic phorbol esters (e.g., prostratin), anti–T cell antibodies (e.g., OKT3), and kinase agonists. It is typically argued that augmenting HAART with deliberate activation should result in the eventual death of all productively infected T cells by a combination of natural mechanisms including viral cytopathic effects, the inherently short life span of activated T cells, and various immune effector mechanisms. Yet to date, trials testing of such approaches have shown no clinical benefit, with at best a reduction in the frequency of latently infected T cells in a subset of patients [2]–[9]. Thus, clinical trials based strictly on flushing out quiescent HIV to purge the infected cell reservoirs have proven disappointing. Further complicating the issue are recent studies suggesting that in most patients, the residual viremia is invariant and genetically distinct from proviruses in resting and activated CD4^+^ T cells; this has led to a hypothesis whereby most of the residual viremia arises from a an unknown cell type, perhaps a stem cell of the monocyte-macrophage lineage, with the capacity for proliferation and continuous release of virus [4].

## Rationale for Targeted Cytotoxic Treatment as a Complement to HAART

Whatever the source(s) and underlying mechanism(s) for the persisting HIV, a major point emphasized herein is that all drugs in the current HAART arsenal share one major feature: their efficacy results from blocking specific steps of the HIV replication cycle, thus preventing new rounds of infection of naïve cells. What they fail to do, at least directly, is to kill cells that are already infected. The theme to be developed here is straightforward: Why not complement the HAART-induced suppression of HIV replication with a treatment that directly kills infected cells? A direct means of achieving this is based on display of the HIV envelope glycoprotein (Env) on the external surface of productively infected cells, where it can be recognized by a specific binding molecule such as an antibody or a soluble fragment of the CD4 receptor. The Env-targeting moiety can be linked to various types of cytotoxic agents, yielding novel molecules that selectively kill HIV-infected cells. This “magic bullet” concept has been prominent in the cancer field, with consideration given to domains of protein toxins, low MW cytotoxic molecules, and radionuclides as alternative cytotoxic payloads [10], [11]. The first successes came a decade ago, with the US Food and Drug Administration's approval of ONTAK (IL-2 linked to the catalytic domain of diphtheria toxin) for cutaneous T cell lymphoma [12], and Mylotarg (a humanized anti-CD33 monoclonal antibody linked to calicheamicin) for relapsing acute myeloid leukemia in elderly patients [13]. The concept may have a particular advantage for infectious diseases [14], since the targeted molecule is encoded by the pathogen, thereby minimizing side effects encountered in anti-cancer applications associated with killing of normal cells expressing low levels of the targeted human antigen. Of course, selective killing requires that the target antigen of the infecting pathogen be expressed on the surface of the infected cell, raising obvious complexities for applications such as HIV infection that are characterized by the presence of latently infected cells.

## Immunotoxin Approaches against HIV in the Pre-HAART Era

Not long after the recognition of the retroviral nature of the AIDS etiologic agent [15], several groups developed cytotoxic agents targeted to HIV Env using antibodies or soluble CD4 linked biochemically or genetically to effector domains of bacterial or plant protein toxins [16]–[19]. Alternative strategies have been considered whereby toxins are targeted to cellular proteins endogenously expressed on T cells, such as the IL-2 receptor on activated CD4^+^ T lymphocytes [20]–[22] or CD45RO on memory T cells [23].

**Figure 1:**
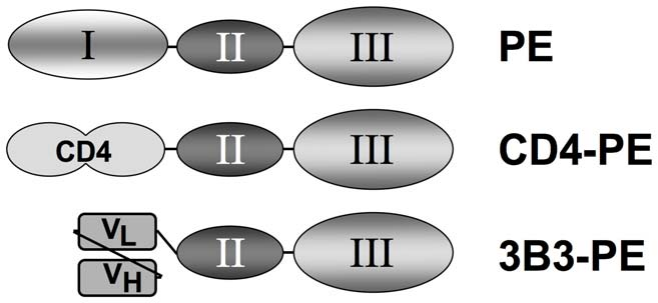
Schematic of Env-targeted toxins based on *Pseudomonas* exotoxin A. The immunotoxins are based on the domain organization of native PE (top). The N-terminal segment (domain I) is involved in binding to a surface receptor (members of the low density lipoprotein receptor-related protein 1 family), the central region (domain II) facilitates membrane translocation of the toxin into the cytoplasm, and the C-terminal segment (domain III) catalyzes ADP-ribosylation of elongation factor 2, resulting in shut-down of protein synthesis and cell death. In the recombinant single chain immunotoxins, domain I is replaced by a targeting moiety directed at HIV-1 gp120: soluble CD4 first two domains (CD4-PE, middle), or the 3B3 SCFv (3B3-PE, bottom).

Over the past two decades, our research groups have collaborated to develop HIV Env-targeted toxins based on *Pseudomonas aeruginosa* exotoxin A (PE). Discrete structural domains within the linear sequence of PE are associated with specific functions [24]. This domain organization has been exploited for cancer therapy by engineering recombinant immunotoxins in which the native N-terminal cell binding domain is replaced by an antibody fragment (typically a single chain SCFv or a disulfide-linked variable region construct) directed against an antigen overexpressed on the specific malignant cell type of interest [25]. To apply this strategy to HIV (Figure 1), we first designed CD4(178)-PE40 (hereafter referred to as CD4-PE) in which the targeting moiety is the first two domains of CD4, which binds directly to the gp120 subunit of Env [16]. Specific cytotoxicity against Env-expressing cells was demonstrated in two types of in vitro systems: a) direct killing assays, in which Env-expressing cells (either stable transfectants or constitutively HIV-infected cell lines) were potently killed in dose-dependent fashion, whereas the corresponding parental cells lacking Env were unaffected [16], [26]–[28], and b) spreading infection inhibition assays, in which infectious HIV-1 is added to permissive target cells, and virus production is measured (p24 or reverse transcriptase) [27], [29]–[32]. CD4-PE inhibited at concentrations where minimal effects were observed with sCD4 (alone or linked to a PE moiety containing an inactivating mutation), thereby demonstrating that the observed activities were due to selective killing of infected cells rather than merely to virus neutralization by the sCD4 moiety. Spreading infection of primary isolates was inhibited [32]–[34] in primary cell types especially relevant to in vivo infection, i.e., peripheral blood mononuclear cells and monocyte-derived macrophages [31], [32], [34]. The latter are particularly noteworthy in view of their extremely low levels of surface Env, as well as the postulated role of macrophages in HIV persistence during HAART given their relatively slow decay kinetics and refractoriness to HIV-mediated cytopathic effects [35], [36].

Based on these promising in vitro findings, CD4-PE was tested in Phase 1 clinical trials in the pre-HAART era [37], [38]. No antiviral or immune-enhancing effects were observed at the maximum tolerated dose of 10–15 µg/kg, which was well below the 40 µg/kg ×3 doses typically given for PE-based cancer immunotoxins. The major dose-limiting toxicity was reversible hepatocellular injury. These disappointing results with CD4-PE greatly diminished enthusiasm for immunotoxins against HIV, and no additional clinical trials have been conducted since.

## Immunotoxin Approaches against HIV: Why Now?

The failed clinical trials with CD4-PE were conducted in the pre-HAART era and thus essentially represented monotherapy (although some individuals also received nucleoside reverse transcriptase (RT) inhibitors that failed to suppress viral loads [37]). The development of HAART prompted us to suggest reconsideration of the Env-targeted toxin concept [39]. In the present report, we propose that experimental and technical advances in the ensuing decade have made this argument even more compelling in several critical ways: a) the persistence of HIV in the face of highly suppressive HAART reveals the need for approaches to augment the depletion of infected cell reservoirs, b) experiments in vitro and in animal models reveal a crucial point: the immunotoxins have limited efficacy in blocking spreading HIV infection when used alone; however, they show dramatic synergistic activities when used in combination with HIV replication inhibitors (discussed below), c) new methods are available to assess various efficacy parameters upon complementing HAART with immunotoxins, d) clinical trials with PE-based immunotoxins against certain leukemias have shown impressive results [40], [41], and e) we have developed an improved immunotoxin with greatly enhanced potency and minimal hepatoxicity potential.

We designed a second PE-based immunotoxin, 3B3(Fv)-PE38 (hereafter referred to as 3B3-PE) [27]. The targeting moiety is the 3B3 SCFv, an affinity-maturated variant of Fab b12 directed against the highly conserved CD4 binding site on gp120; compared to b12, 3B3 displays improved binding affinity and greater breadth of reactivity against Envs from HIV-1 primary isolates [42]. We compared the potencies of the two PE-based immunotoxins in several in vitro systems. In direct cell killing assays against Env-expressing cell lines, 3B3-PE displayed significantly enhanced potency (IC_50_ 0.03–0.04 nM) compared to CD4-PE (IC_50_ 0.6–1.5 nM); neither agent was cytotoxic against the corresponding Env-negative cell lines [28]. Both immunotoxins inhibited spreading infection of all the HIV-1 primary isolates tested (clade B), again with 3B3-PE showing greater potency than CD4-PE [43]. Like the original immunotoxin, 3B3-PE inhibited HIV-1 spreading infection in monocyte-derived macrophages. Most importantly, an extremely high intravenous dose of 3B3-PE (250 µg/kg ×3) caused no hepatotoxicity in rhesus macaques, in contrast with the elevation of serum hepatic enzymes induced by CD4-PE at the same dosage [43]. We had previously speculated [39] that the dose-limiting hepatotoxicity observed in the CD4-PE Phase 1 trials might have been due to the CD4 moiety of the chimeric toxin binding to free gp120 released from virions and infected cells, leading to nonspecific liver uptake perhaps via the asialoglycoprotein receptor on hepatocytes recognizing oligosaccharide chains on gp120. Subsequent studies argue against this hypothesis. First, the distinct macaque hepatotoxicity profiles noted above (none for 3B3-PE; significant for CD4-PE) have also been seen in simian human immunodeficiency virus (SHIV)-infected animals that expressed very high viral loads (W. Wagner, M. G. Lewis, E. A. Berger, and I. Pastan, unpublished data); if hepatotoxicity reflected binding to released gp120, both chimeric toxins should have caused similar effects. Second, animal studies with other PE-based immunotoxins have revealed that hepatoxicity is associated with a high isoelectric point of the Fv [44]; the highly basic nature of the CD4 moiety (isoelectric point 8.86) likely underlies the hepatotoxicity of CD4-PE. We conclude that 3B3-PE is a much more promising agent than CD4-PE, with a significantly improved therapeutic window due to its enhanced specific cytotoxic potency and greatly reduced likelihood for dose-limiting hepatotoxicity.

## Potent Synergy between Immunotoxins and Inhibitors of HIV Replication

**Figure 2:**
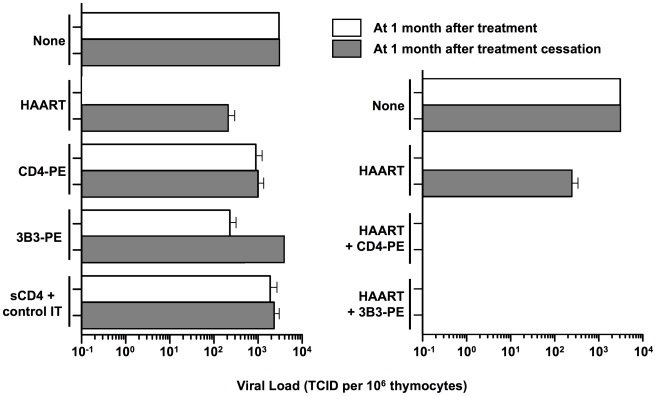
Effects of complementing HAART with Env-targeted toxins in thy/liv SCID-hu mouse model. Thy/liv-SCID-hu mice were injected intraperitoneally with HIV and either left untreated (None) or immediately started on the indicated treatment. One month later, the left thy/liv implants were biopsied, and viral loads were analyzed by quantitative coculture (*open bars*). Drug therapy was then stopped, and after 1 month the left thy/liv implant of each mouse was rebiopsied and the viral load quantitated (*filled bars*). The data are presented as the TCID/10^6^ thymocytes; the mean values (± SEM) were calculated for each group. Left panel, HAART (zidovudine+ lamivudine + ritonavir) or immunotoxins alone; right panel, HAART alone compared to HAART plus immunotoxins. Data adapted from [45] (© 2000 by Goldstein et al.).

Our cell culture studies demonstrated significant activity of CD4-PE and 3B3-PE against spreading HIV-1 infection. However, we observed limitations that we now view as critical for our current thinking about how immunotoxins should be considered for clinical use. First, while both CD4-PE and 3B3-PE inhibited spreading infection, the effects were relatively inefficient compared to activities in direct killing assays; at best the viral peak was reduced and delayed and the killing of target cells was slowed, but the effects were never complete [30], [43]. Moreover, the IC_50_ values in spreading infection assays were significantly weaker than in direct killing assays against target cells uniformly expressing Env. These findings can be readily understood in terms of the mode of action of these agents; they cannot kill a newly infected cell until surface Env is expressed, by which time the virus infection has already begun to spread. Therefore it can be predicted that the presence of replication inhibitor would render immunotoxin action more similar to what is observed in direct killing assays, i.e., greater potency and complete activity. Indeed, early cell culture studies showed marked synergy between RT inhibitors and CD4-PE, with the former agents dramatically reducing the IC_50_ values of the immunotoxin (and vice versa); combination treatment completely eradicated HIV-1 from the culture [30]. Subsequent experiments in the SCID-hu (thy,liv) mouse model gave parallel results [45], as shown in Figure 2. Combination of replication inhibitors (zidovudine plus lamivudine plus ritonavir) alone greatly suppressed HIV levels in the human tissue implant after a 30-day treatment period, but the loads rebounded as measured at 30 days after cessation of treatment; CD4-PE or 3B3-PE alone only minimally suppressed viral loads at the end of the treatment period, and at 30 days post-treatment cessation. The results were strikingly different with the combination of HAART plus either CD4-PE or 3B3-PE: viral loads were strongly suppressed not only at the end of the 30-day treatment period, but also at 30 days after treatment cessation. These results highlight the particular value of combining HAART drugs, which potently block HIV replication, with Env-targeted toxins, which kill cells that are already infected.

## The Way Forward

Based on the considerations outlined above, we believe the time has come for clinical trials of an Env-targeted cytotoxin as a means to deplete infected cell reservoirs persisting in the face of suppressive HAART. This proposal differs fundamentally from HAART “intensification”, whereby a new HIV replication inhibitor is added to an already suppressive antiretroviral regimen. Instead, it represents “complementation” of one class of agents that blocks viral replication (HAART drugs) with a second class that kills those cells already infected (immunotoxin).

“Proof-of-concept” preclinical studies can be conducted in nonhuman primate models of HIV therapy and persistence [46]–[50]. Of particular interest is an Env-SHIV model in which CD4^+^ T lymphocytes are rapidly depleted, but high viral loads are generated from infected tissue macrophages that appear to be refractory to the viral cytopathic effects and resistant to the antiviral activity of the nucleoside RT inhibitor PMPA [47]. Would immunotoxin treatment deplete this pool of virus-producing cells? Perhaps more relevant to conditions under which immunotoxins might be used in humans are the recently developed chimeric SIVs harboring the HIV-1 reverse transcriptase (RT-SHIVs) [46], [48], [49]. This model permits testing of the same nucleoside and nonnucleoside RT inhibitors currently used in modern HAART regimens. However, the SIV Envs in such SHIVs are not reactive with the 3B3 antibody, thus precluding analysis of 3B3-PE; the alternative CD4-PE could be examined, since it is active against SIV Envs [27] and would cause negligible hepatotoxicity in macaques at the doses that would be employed (40 µg/kg, ×3, unpublished data). While such studies might prove interesting, it is presently unclear whether any of the macaque models will faithfully replicate the mechanisms of HIV persistence in humans, thus arguing against delaying immunotoxin clinical trials until macaque efficacy studies are performed.

3B3-PE is the agent of choice for Phase 1 clinical trials of immunotoxin complementation of HAART, due to its likely improved therapeutic window compared to CD4-PE. The most straightforward approach would involve a one-two punch, i.e., administration of 3B3-PE to patients whose plasma viremia has been well suppressed by HAART. Would persisting HIV be lowered significantly, perhaps to undetectable levels (unlike what has been reported recently for residual viremia with HAART intensification using additional replication inhibitors [51], although contradictory findings with the integrase inhibitor raltegravir have been presented recently [52], [53])? The potential impact of immunotoxin complementation is particularly intriguing in view of the hypothesis noted above that residual viremia may derive predominantly from an unknown cell type capable of proliferation and continuous virus release [54], [55]; immunotoxin treatment might be uniquely suited to target such virus-producing cells. However, the fact that immunotoxin activity is restricted to cells expressing surface Env is almost certain to compromise its ultimate efficacy in the context of persisting reservoirs of latently infected CD4^+^ T cells. Thus, durable benefits might require a three-tiered attack, i.e., combining HAART plus immunotoxin with a treatment to deliberately trigger HIV expression from latent proviral genomes. In vitro and ex vivo experiments have documented the ability of CD4-PE [26] and 3B3-PE [56] to selectively kill latently infected CD4^+^ T cells after induction of virus expression; similarly, activating agents have been shown to increase the immunotoxin susceptibility of cultured macrophages [57]. Given the limited understanding and conflicting viewpoints regarding the mechanisms underlying HIV persistence and rebound, we believe it is essential to remain open-minded regarding the testing of two-tiered versus three-tiered approaches. In fact, augmenting HAART with candidate HIV-inducing regimens and immunotoxins, separately and in combination, might provide mutually informative insights. For example, it is possible that an inducing regimen deemed minimally effective in previous clinical trials might have had benefits that went unnoticed in the absence of targeted killing of the newly activated infected cells; conversely, the duration of immunotoxin-mediated benefits might be negligible without prior purging of latently infected cells.

3B3-PE treatment will presumably be limited to short periods (probably three intravenous doses weekly for 1–2 weeks, based on cancer protocols), since the immunotoxin will likely elicit activity-blocking antibodies against the highly immunogenic PE moiety (as was observed in the CD4-PE Phase 1 trial [37]). Should promising results be obtained and additional treatment be desired, the potential exists for switching to Env-targeted cytotoxins based on alternate bacterial or plant protein toxin moieties as noted above, or newly described agents employing low molecular weight drugs [58] or radionuclides [59] as the cytotoxic payloads. Efforts to delete B cell epitopes from PE38 [60] may also prove useful in this context. Regarding enhancements and variations of the targeting moiety, structure-based design has been used to improve the potency of 3B3-PE [61]. However, it must be noted that not all HIV-1 primary isolates are susceptible to neutralization by the 3B3 antibody [42]; thus, it will be important to assess for each potential clinical trial participant whether their virus is recognized by 3B3 (using virus and/or cells obtained from CD8-depleted activated peripheral blood mononuclear cells). The possibility must also be considered for using alternate immunotoxins containing different targeting moieties, such as those described against gp41 [62], [63] or new immunotoxins based on well-studied [64] or newly described [65] broadly neutralizing monoclonal antibodies.

How might immunotoxin efficacy be assessed? Beyond analysis of peripheral blood (proviral DNA and infectious virus in CD4^+^ T cells; residual viremia detected by single copy assay), the profound involvement of gut-associated lymphoid tissue during both acute HIV infection and viral persistence under HAART [66] highlights the critical importance of examining this compartment. Indeed, HIV DNA levels have been shown to be highly elevated in gut biopsy tissue compared to those in peripheral blood from patients on HAART [67].

Ultimately, immunotoxins will be of value in HIV treatment only if they can enable patients to stop HAART for prolonged periods, sufficient to provide a meaningful quality of life benefit. This is a stringent demand in view of the ongoing improvements in the efficacy and acceptability of antiretroviral drugs, and the risks associated with intermittent cessation of HAART [68]. The ultimate question underlying the potential value of immunotoxins is: must every last infected cell in the body be eliminated in order to achieve a meaningful therapeutic benefit, or is it possible that the infected cell load can be reduced below a threshold such that natural immune effector mechanisms, perhaps enhanced by therapeutic vaccines, can keep the infection in check without the need for ongoing drug treatment? Only a focused effort on clinical evaluation, with all its associated complexities, will provide an answer.
